# Multiscale Information Transfer in Functional Corticomuscular Coupling Estimation Following Stroke: A Pilot Study

**DOI:** 10.3389/fneur.2018.00287

**Published:** 2018-05-01

**Authors:** Xiaoling Chen, Ping Xie, Yuanyuan Zhang, Yuling Chen, Fangmei Yang, Litai Zhang, Xiaoli Li

**Affiliations:** ^1^Key Laboratory of Measurement Technology and Instrumentation of Hebei Province, Institute of Electric Engineering, Yanshan University, Qinhuangdao, China; ^2^Institute of Education Science, Applied Psychology of Tianjin Province, Tianjin Normal University, Tianjin, China; ^3^Department of Rehabilitation Medicine, The NO.281 Hospital of Chinese People’s Liberation Army, Qinhuangdao, China; ^4^National Key Laboratory of Cognitive Neuroscience and Learning, Beijing Normal University, Beijing, China

**Keywords:** functional corticomuscular coupling, multiscale, transfer entropy, stroke, information flow

## Abstract

Recently, functional corticomuscular coupling (FCMC) between the cortex and the contralateral muscle has been used to evaluate motor function after stroke. As we know, the motor-control system is a closed-loop system that is regulated by complex self-regulating and interactive mechanisms which operate in multiple spatial and temporal scales. Multiscale analysis can represent the inherent complexity. However, previous studies in FCMC for stroke patients mainly focused on the coupling strength in single-time scale, without considering the changes of the inherently directional and multiscale properties in sensorimotor systems. In this paper, a multiscale-causal model, named multiscale transfer entropy, was used to quantify the functional connection between electroencephalogram over the scalp and electromyogram from the flexor digitorum superficialis (FDS) recorded simultaneously during steady-state grip task in eight stroke patients and eight healthy controls. Our results showed that healthy controls exhibited higher coupling when the scale reached up to about 12, and the FCMC in descending direction was stronger at certain scales (1, 7, 12, and 14) than that in ascending direction. Further analysis showed these multi-time scale characteristics mainly focused on the beta1 band at scale 11 and beta2 band at scale 9, 11, 13, and 15. Compared to controls, the multiscale properties of the FCMC for stroke were changed, the strengths in both directions were reduced, and the gaps between the descending and ascending directions were disappeared over all scales. Further analysis in specific bands showed that the reduced FCMC mainly focused on the alpha2 at higher scale, beta1 and beta2 across almost the entire scales. This study about multi-scale confirms that the FCMC between the brain and muscles is capable of complex and directional characteristics, and these characteristics in functional connection for stroke are destroyed by the structural lesion in the brain that might disrupt coordination, feedback, and information transmission in efferent control and afferent feedback. The study demonstrates for the first time the multiscale and directional characteristics of the FCMC for stroke patients, and provides a preliminary observation for application in clinical assessment following stroke.

## 1 Introduction

Motor dysfunction is a major consequence of stroke (1), and the loss of motor function is generally considered as a result of the impairments in neural network that controls movement. Therefore, an effective and precise assessment on the motor functions of stroke patients plays an important role in motor recovery.

The functional corticomuscular coupling (FCMC) between the motor cortex and the effector muscles is considered essential for effective movement control (2). Extensive studies have expounded that cortical oscillatory drives are coupled with muscle activation in several different frequency bands. Corticomuscular oscillations in alpha-band (8–14 Hz) have been reported during sustained contractions (3), slow finger movements (4, 5), and fast transitions between two force targets (6). Beta-band oscillations (15–35 Hz) are associated with strategies for controlling and maintaining steady-state force output (6–11). Oscillations in the gamma-band (35–60 Hz) are related to stronger muscle force production (12, 13) and dynamic force output (6, 14). These researches reveal that the FCMC in different frequency bands plays different roles in sensory and motor systems in healthy subjects.

Similar studies have been carried out on the stroke patients, since Mima et al. (15) first reported that the FCMC for the hand and forearm muscles was smaller on the affected side of subcortical stroke patients during weak tonic contraction tasks. Fang et al. (16), Meng et al. (17), and von Carlowitz-Ghori et al. (18) also reported that stroke patients had significant lower FCMC on affected sides during a steady-state force task. FCMC decrease indicates that the impairment in the lesioned hemisphere possibly leads to the discontinuity of information transmission in the sensory-motor systems. However, Braun et al. (19) concluded a conversely preliminary observation that maximal FCMC in some patients with excellent recovery were higher than that in the healthy controls during a steady grip task, and Graziadio et al. (20) reported that there were no FCMC differences between stroke patients and healthy controls during rest and isometric contraction. The above studies without uniform conclusions mainly focus on the functional coupling and temporal coordination, and also indicate that the FCMC between the motor cortex and the muscle can be considered as an assessment of motor recovery. Several reports, however, point out that the FCMC possibly conveys the central motor command (descending), but not the sensory afferent feedback (ascending) for stroke patients suffering from a pure motor paresis without sensory symptoms (15). The direction-dependent information transmission between the brain and the muscle thereby seems to be necessary to analyze inherent mechanism for stroke.

Unfortunately, only a few studies of the FCMC in information flow are carried out in healthy people. These researches have shown that the cortical oscillations between the cortex and the muscle are direction-dependent (21, 22). Witham et al. (23) found that the FCMC strength was larger in descending pathway than that in ascending pathway in monkey, and they (24) also revealed that the FCMC in ascending pathway was dominated within the whole beta-band (12–30 Hz) compared with that in descending pathway in humans. Mima et al. (25), however, reported that the FCMC in descending direction was significantly larger at 19–30 Hz band than that in the opposite direction. Although these works indicate that the FCMC can elucidate functionally relevant contributions of cortical oscillation to motor control and muscle activation to sensory feedback, it is hard to centralize uniform conclusions. These inconsistent results may be from the applied methodologies limited to focus on the linear or nonlinear assessment of the functional coupling, interaction strength, and information flow. However, these methods cannot be utilized to describe the multiscale characteristics of complex electroencephalogram (EEG) and electromyogram (EMG) series, whereas the brain function is regulated by complex self-regulating systems that process inputs from interacting mechanisms which operate in multiple spatial and temporal scales (26). Therefore, it is necessary to go into research on methods that can analyze more information in motor-control system.

Transfer entropy (TE) technique (27) as a causal tool can measure the effective connectivity and capture nonlinear nature based on information entropy without modeling the interaction. Based on the asymmetry (reflecting directional) and transition (reflecting dynamic) probabilities computation, the TE method is particularly efficient in detecting some unknown nonlinear interactions (28) and has been applied into neuroscience (28–31). Therefore, the TE is suitable in analyzing the functional connections between the cortex and the contralateral muscles in sensorimotor loop system. The measure, however, is still single-scale and may be insufficient to describe the dynamical and multiscale characteristics of complex EEG and EMG series. In our previous study, we have proposed multiscale transfer entropy (MSTE) by introducing the coarse-graining process into the TE method (32). This study revealed the temporal-scale characteristics in FCMC based on the analysis between the EEG and EMG signals in healthy controls. However, there was no similar analysis in stroke patient, though large studies about the EEG signal for stroke have indicated a decreased complexity of the neural activity in the brain (33–35). Similar to the brain, we guess that the spatial and temporal scales in the sensorimotor system may be disordered for stroke patients due to the structural lesion in the cerebral brain.

The main contribution of this work is the study of the multiscale and directional characteristics of the FCMC between the cerebral cortex and the contralateral muscles for stroke. The MSTE method was applied to experimental data recorded while performing the grip task with steady-state force in stroke patients and healthy controls. Such studies can provide new insight into the dynamical and multiscale characteristics of functional connections in coupling strength and coupling flow after stroke and add to the understanding of mechanisms underlying motor recovery. The present study demonstrates for the first time the multiscale characteristics of the FCMC between the brain and the contralateral muscles in both pathways for stroke patients.

## 2 Materials and Methods

### 2.1 Subjects

8 stroke patients who had persistent dyscoordination of the right upper limb without sensory symptoms (Table 1; mean age, 52.6 ± 9.6years; range, 37–66 years; 3 male) and 8 healthy controls (mean age, 59.4 ± 6.2 years; range, 53–69 years; 5 male) without any history of neurological disease were enrolled in the study. The participants were tested according to the Oldfield questionnaire (36). All subjects participated according to the declaration of Helsinki and gained consent and approval of the ethical review board of Yanshan University. All participants have given informed consent. They all had no previous experience with similar experiments.

**Table 1:** Demographic information of stroke patients.

Patients	Age	Months since stroke	STM for affected side	Lesion site	Stroke type
1	40–45	5	8	R Periventricular	Ischemia
2	50–55	15	4	R Temporal lobe, external capsule	Ischemia
3	36–40	6	6	L Frontal lobe, centrum semiovale, periventricular	Ischemia
4	50–55	10	6	Pons	Ischemia
5	46–50	12	8	R Basal ganglia	Hemorrhage
6	50–55	8	9	Pons	Ischemia
7	55–60	13	7	R Basal ganglia	Hemorrhage
8	55–60	11	5	Pons	Hemorrhage

*R, right hemisphere; L, left hemisphere; STM, Shang tian min functional clinical assessment scale*.

### 2.2 Data Recording and Experiment Paradigm

#### 2.2.1 Experimental Paradigm

**Figure 1:**
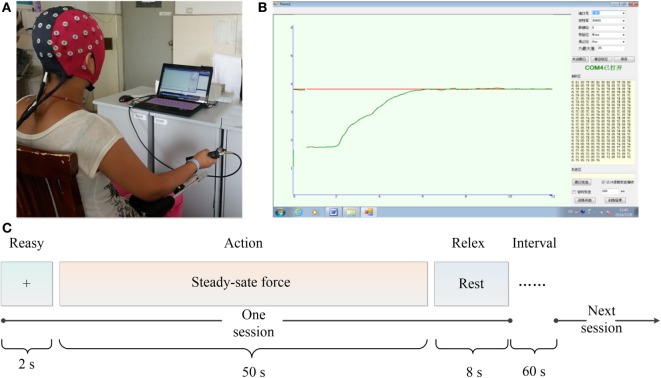
Experimental setup. **(A)** Recording of electroencephalogram and electromyogram data. **(B)** Force profile generated by the mainpulandum during 1 trial: the red line indicated the target force and the green line represented the exerted force. **(C)** The flow of the experimental task.

During the experimental session, the subjects sat in an electrically shielded, dimly lit room. All subjects were instructed to place their right-hand to grip a shank which connected to a force sensor (Figure 1A). Visual feedback on the force were provided for the subjects *via* a monitor with two lines in different colors: the red line indicated the target force (TF) and the green line represented the exerted force (EF) by the subjects. The subjects needed to maintain the green line tracking the red line at any time (Figure 1B).

The total experiment mainly contained two sections as shown in Figure 1C. First, the subjects were asked to perform the maximum voluntary contraction (MVC). Before the task, each subject performed an isometric contraction of the right-hand grip with maximal effort lasting 5 s and the MVC force was determined as the peak value over the period of stable force output. To obtain precise result, the MVC was measured three times for each subject and calculated the mean value as $M\tilde{V}C$. The whole task included four sessions with 60 s break between each session to avoid fatigue, and each session with 2 s ready, 50 s steady-state force output with 20% $M\tilde{V}C$, and 8 s relax.

#### 2.2.2 EEG and EMG Data Recording

During the experiments, scalp EEG and EMG signals were recorded synchronously by 64-channel eego™ sports system (ANT Neuro, Enschede, Netherlands) and 1-channel Trigno™ Wireless EMG system (Delsys Inc., Natick, MA, USA). These two sets of equipment were combined by a wireless synchronous pulse trigger. EEG signals were recorded from 32 scalp positions using the international 10–20 system and the EMG signals were recorded from the flexor digitorum superficialis (FDS). Before the electrode application, the hair needed to clean and dry off, and the skin surface was cleaned with alcohol. The EEG and EMG data were amplified (1,000) and digitized (1,000 Hz).

#### 2.2.3 Data Preprocessing

Data analysis was carried out offline in MATLAB (R2013b, Mathworks Inc., Natick, MA, USA) environment. In order to avoid the impact of the beginning force, the interval between 2 and 48 s after the tone onset was chosen for further analysis. Segments with large-amplitude artifacts in the range of 0.5–150 Hz were excluded. And the corresponding EMG and EEG signals of those epochs were discarded, too. After visual inspection, we designed a combined filter to remove the artifacts in raw EEG recordings. First, mean and SD rejected outlier points. Then, an adaptive notch filter (37) was used to remove the 50 Hz the power signal, and a high-pass filter was used to remove baseline drift. After that, Informax-based independent component analysis (ICA) was used to remove the electrooculogram (EOG) signals. Finally, canonical correlation analysis was implemented to remove the EMG signal from the EEG signal (38). A bipolar derivative (39) was analyzed for MSTE calculation in the subsequent analysis. Compared to EEG signals, the interferences in EMG signals were easily removed. An adaptive notch filter was used to remove the 50 Hz power signal, and a 0.5–150 Hz bandpass filter was used to remove the direct current high frequency interference. After that, the EMG signals were rectified before subsequent analysis (40, 41).

### 2.3 Multiscale TE

#### 2.3.1 Derivation of multiscale TE

To account for the inherent multiscale characteristics in the brain or the muscle, a “coarse graining” process was applied. *X* = {*x*_1_, *x*_2_,…,*x_i_*,…, *x_N_*} and *Y* = {*y*_1_, *y*_2_,…,*y_i_*,…, *y_N_*} represented the preprocessed EEG and EMG signals; {*x^s^*} and {*y^s^*} were constructed consecutive coarse-grained time series by averaging *X* and *Y* data points in non-overlapping windows of length, respectively. Each element of the coarse-grained time series was calculated according to the equations:

(1) $$\begin{array}{lll} x_{j}^{\left(s\right)}=1∕s\sum_{i=(j-1)s+1}^{js}x_{i}\left(1\leq j\leq N∕s\right) & \; & \end{array}$$

(2) $$\begin{array}{lll} y_{j}^{\left(s\right)}=1∕s\overset{\mathrm{js}}{\underset{i=\left(j-1\right)s+1}{\sum }}y_{i}\left(1\leq j\leq N∕s\right) & \; & \end{array}$$

where *s* represents the scale factor, and the $x_{j}^{\left(s\right)}$ and $y_{j}^{\left(s\right)}$ denote the element of the coarse-grained time series {*x^s^*} and {*y^s^*}. The length of each coarse-grained time series is equal to the length of the original time series *N* divided by *s*. For scale *s* = 1, the time series {*x*^1^} and {*y*^1^} are the original EEG and EMG series, respectively. Next the TE was calculated for each coarse-grained time series {*x^s^*} and {*y^s^*}. The TE values from *X^s^* to *Y^s^*, termed ${\text{MSTE}}_{\text{EEG}\to \text{EMG}}^{s}$, can be derived from conditional entropies as follows:

(3) $${\text{MSTE}}_{\text{EEG}\to \text{EMG}}^{s}=H^{s}\left(y_{t+u}\left|y_{t}^{n}\right.\right)-H^{s}\left(y_{t+u}\left|x_{t}^{m}\right.,y_{t}^{n}\right)$$

where *t* is a discrete valued time-index and *u* is a scalar value, namely the information transfer delay between *X^s^* and *Y^s^*; $x_{t}^{m}=\left(x_{t},\cdot \cdot \cdot ,x_{t-m+1}\right)$ and $y_{t}^{n}=\left(y_{t},\cdot \cdot \cdot ,y_{t-n+1}\right)$ are *m*- and *n*- dimensional delay vectors of *X^s^* and *Y^s^*, respectively; $H^{s}\left(y_{t+u}\left|y_{t}^{n}\right.\right)$ is the entropy of the process *Y^s^* conditional on its past, and can be calculated as

(4) $$H^{s}\left(y_{t+u}\left|y_{t}^{n}\right.\right)=-\underset{y_{t+u}}{\sum }p\left(y_{t+u},y_{t}^{n}\right)\log_{2}\left(p\left(y_{t+u}∕y_{t}^{n}\;\right)\right)$$

$H^{s}\left(y_{t+u}\left|x_{t}^{m}\right.,y_{t}^{n}\right)$ can be also calculated as

(5) $$H^{s}\left(y_{t+u}\left|x_{t}^{m}\right.,y_{t}^{n}\right)=-\underset{y_{t+u}}{\sum }p\left(y_{t+u},y_{t}^{n},x_{t}^{m}\right)\log_{2}\left(p\left(y_{t+u}∕y_{t}^{n},x_{t}^{m}\;\right)\right)$$

where the formula (3) can be rewritten as

(6) $$\begin{array}{llll} & {\text{MSTE}}_{\text{EEG}\to \text{EMG}}^{s}=\underset{y_{t+u},y_{t}^{n},x_{t}^{m}}{\sum }p\left(y_{t+u},y_{t}^{n},x_{t}^{m}\right)\log_{2}\frac{p\left(\left.y_{t+u}\right|y_{t}^{n},x_{t}^{m}\right)}{p\left(\left.y_{t+u}\right|y_{t}^{n}\right)}\; & & \;\\ & \;=\underset{y_{t+u},y_{t}^{n},x_{t}^{m}}{\sum }p\left(y_{t+u},y_{t}^{n},x_{t}^{m}\right)\log_{2}\frac{p\left(y_{t+u},y_{t}^{n},x_{t}^{m}\right)p\left(y_{t}^{n}\right)}{p\left(y_{t+u},y_{t}^{n}\right)p\left(y_{t}^{n},x_{t}^{m}\right)}\; & \end{array}$$

The MSTE from *Y^s^* to *X^s^* can be defined as ${\text{MSTE}}_{\text{EMG}\to \text{EEG}}^{s}$, and can be obtained by the same process. The ${\text{MSTE}}_{\text{EEG}\to \text{EMG}}^{s}$ $\mathrm{MST}{\text{E}}_{\mathrm{EEG}\to \mathrm{EMG}}^{s}$ and ${\mathrm{MSTE}}_{\text{EMG}\to \text{EEG}}^{s}$ $\mathrm{MST}E_{\mathrm{EMG}\to \mathrm{EEG}}^{s}$ can be used to describe the information flow across the whole bands from the EEG to EMG and from EMG to EEG, respectively. In this paper, the MSTE values will be calculated between each pair of EEG–EMG, and this procedure may account for the different locations of the maximum MSTE values due to inter-individual differences in brain morphology. For each EEG channel, only the higher MSTE values were used for subsequent analysis.

#### 2.3.2 Statistical Significance

To test the statistical significance of the values for MSTE at each time scale, we used the surrogate data method by randomizing the phase of the original data which can obtain by Fourier transform (42). This will destroy the causal interaction, but guarantee the same amplitude characteristics between the surrogate data and the original data. For each signal *X^s^* or *Y^s^* in each time scale, we performed 10,000 times and calculated the MSTE in both directions at each time scale, respectively. After that, the mean values across all 10,000 times, named $\text{M}\tilde{\text{S}}\text{TE}$, were calculated in each direction at each scale. Therefore, if the MSTE values was larger than the $\text{M}\tilde{\text{S}}\text{TE}$ in the same direction and time scale, we can conclude there was significant causal interaction. In the subsequent analysis, we calculated the difference values by the MSTE values subtracting the $\text{M}\tilde{\text{S}}\text{TE}$ values, and we defined as zero if the difference value was negative.

### 2.4 Statistical Analysis

To investigate the differences between stroke patients and healthy controls in both directions, three-way repeated measures analysis of variance (rANOVA) (43, 44) was performed with subject (2 levels: stroke and control) as a between-subject factor, direction (2 levels: descending and ascending), and time scale (20 scales) as within-subject factors, and the MSTE value as the dependent variable. In the case of significant subject by direction interaction, simple effect was used to compare the subject differences on a level of the direction or the direction differences on a level of the subject separately for each time scale. In our study, to describe the corticomuscular interaction in specific frequency bands, we used the wavelet package method (45) to divide the EEG and EMG signals into sub-bands, and then reconstituted the specific frequency bands, such as delta (1–4 Hz), theta (4–8 Hz), alpha1 (8–10 Hz), alpha2 (10–12 Hz), beta1 (12–25 Hz), beta2 (25–35 Hz), gamma1 (35–45 Hz), and gamma2 (45–60 Hz). In each specific frequency band, we then calculated the MSTE values. After that, in each sub-band, we also performed three-way rANOVA with subject (2 levels: stroke and control) as a between-subject factor, direction (2 levels: descending and ascending), and time scale (20 scales) within-subject factors. In the case of significant subject by direction interaction, simple effect was also used to compare the subject differences on a level of the direction or the direction differences on a level of the subject separately for each time scale. Greenhouse–Geisser was used to correct the degree of freedom. In this study, an alpha of *P* < 0.05 was considered significant. SPSS 19.0 for windows (SPSS Inc., Chicago, IL, USA) was used for all statistical computations.

## 3 Results

### 3.1 MSTE Values for SP and HC

**Figure 2:**
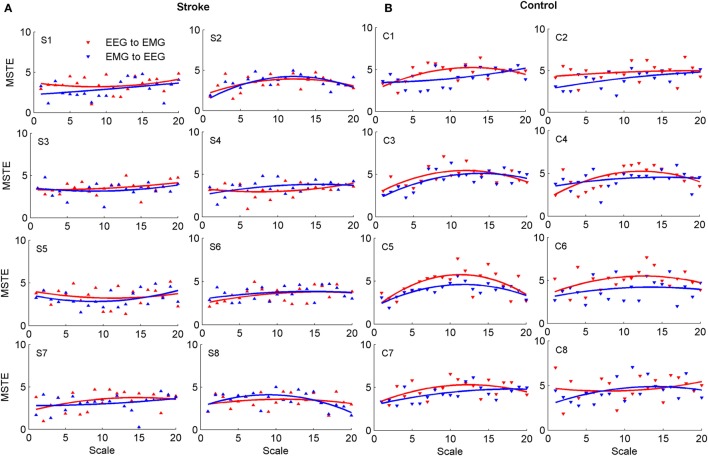
The MSTE values in descending and ascending directions for both stroke patients and healthy controls, respectively. **(A)** Showed the MSTE values in two directions for each stroke patient and **(B)** for each healthy control. The label “S” in **(A)** represented the stroke patient, and the label “C” in **(B)** meant the healthy control.

**Figure 3:**
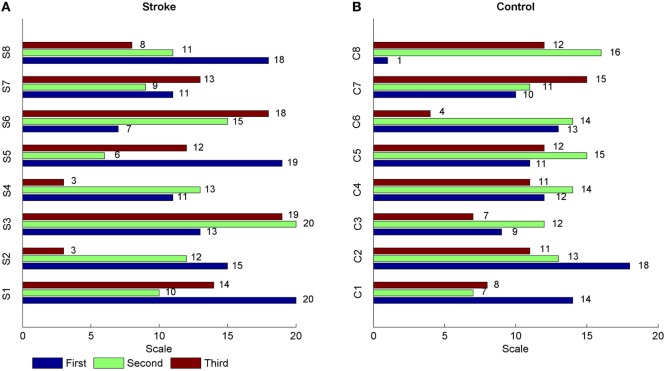
The scales distribution where the first three higher MSTE values located for each subject. The label “S” in **(A)** represented the stroke patient, and the label “C” in **(B)** meant the healthy control.

Figure 2 showed the MSTE values in descending and ascending directions for both stroke patients and healthy controls as the scale increased. Figure 2A showed the MSTE values in two directions for each stroke patient and Figure 2B for each healthy control. As Figure 2B was shown, for healthy controls, the interaction strength had a gradually increasing trend even if the growth rate was declined in both two directions as the scale *s* increased, and reached a steady state when the scale reached up to 12 even if the scale increased. Compared to the healthy controls, Figure 2A showed that there were some commons between stroke patients and healthy controls that the strengths also increased even if the growth rate was declined with the scale increasing. However, there was no steady state during the scale interval from 1 to 20, and even a significant decline in ascending direction for stroke patient S8. What is more, we plotted the scales where the first three higher MSTE values located in Figure 2. As Figure 3 was shown, for healthy controls, the scale mainly focused on about 12, except for the ascending direction in C2 and descending direction in C8. However, there was no regular difference in both directions for stroke patients. Further comparison between the descending and ascending directions showed that the MSTE values in descending direction for most healthy controls were higher at the scale about 5~15 than that in ascending direction. However, we cannot find similar differences between two directions for stroke patients, except for S8.

### 3.2 MSTE Values in Each Direction for SP vs. HC

**Figure 4:**
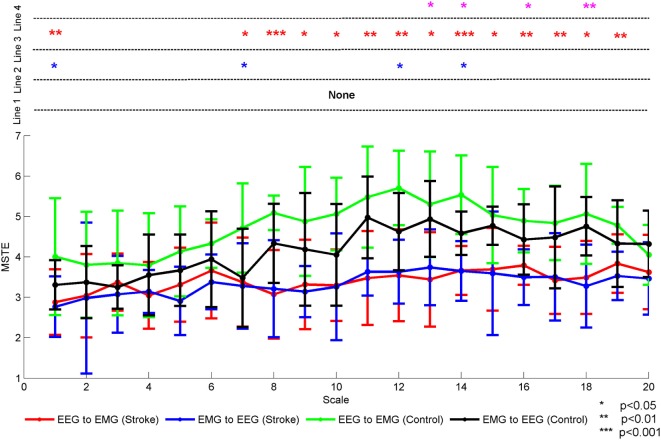
The mean MSTE values in both directions for both stroke patients and healthy controls with the time scale increasing. We denoted the significance with the star mark. *p < 0.05, **p < 0.01, and ***p < 0.001. The word “Line 1” showed the significant level between descending and ascending direction in each time scale for stroke patients, the word “Line 2” was for healthy controls. The Line 3 and Line 4 indicated the significant level between stroke patients and healthy controls at each time scales in descending and ascending directions, respectively.

Three-way rANOVA yielded significant main effects for subject [*F*(1,14) = 390.900, *p* = 0.000], direction [*F*(1,14) = 20.125, *p* = 0.001], and time scale [*F*(8.392,117.488) = 4.424, *p* = 0.000], respectively. There were also a double interaction between the subject and the direction [*F*(1,14) = 10.871, *p* = 0.005]. However, there was no interaction among subject, direction, and time scale [*F*(7.328,102.586) = 0.351, *p* = 0.933]. Further statistical analysis showed the MSTE differences in descending or ascending direction between stroke patients and healthy controls in each time scale, respectively. Figure 4 showed the mean MSTE values across all subjects in two directions with the time scale increasing. In this figure, we denoted the significance with the star mark. The word “Line 1” showed the significant level between descending and ascending direction in each time scale for stroke patients, the “Line 2” was for healthy controls. The Line 3 and Line 4 indicated the significant level between stroke patients and healthy controls at each time scale in descending and ascending directions, respectively. As this figure was shown, there was no significant difference between descending and ascending directions for stroke patients at each time scale. On the contrary, there were significant differences for healthy controls at scale 1 [*F*(1,14) = 4.35, *p* = 0.031], scale 7 [*F*(1,14) = 5.94, *p* = 0.029], scale 12 [*F*(1,14) = 4.85, *p* = 0.045], and scale 14 [*F*(1,14) = 6.99, *p* = 0.019]. This mean the MSTE values in descending direction was significantly larger than those in the opposite direction. Compared to healthy controls, stroke patients lost the difference between the descending and ascending directions. Additionally, we analyzed the differences between the stroke patients and healthy controls in each direction. As Line 3 was shown, healthy controls showed higher MSTE values in descending direction compared to stroke patients at scale 1 [*F*(1,14) = 9.78, *p* = 0.009] and high scales from 7 to 19. Meanwhile, healthy controls also represented significantly higher MSTE values in ascending direction compared to stroke patients at scale 13 [*F*(1,14) = 6.48, *p* = 0.023], scale 14 [*F*(1,14) = 8.42, *p* = 0.012], scale 16 [*F*(1,14) = 5.63, *p* = 0.033], and scale 18 [*F*(1,14) = 11.12, *p* = 0.005], respectively. These differences also meant stroke patients had a decreased coupling in both directions at high time scale. Additionally, we can also find that the healthy controls had a higher MSTE values at scale 12 in both directions, which had a similar distribution as each subject showed in Figure 2.

### 3.3 MSTE Values at Each Frequency Band for SP vs. HC

**Figure 5:**
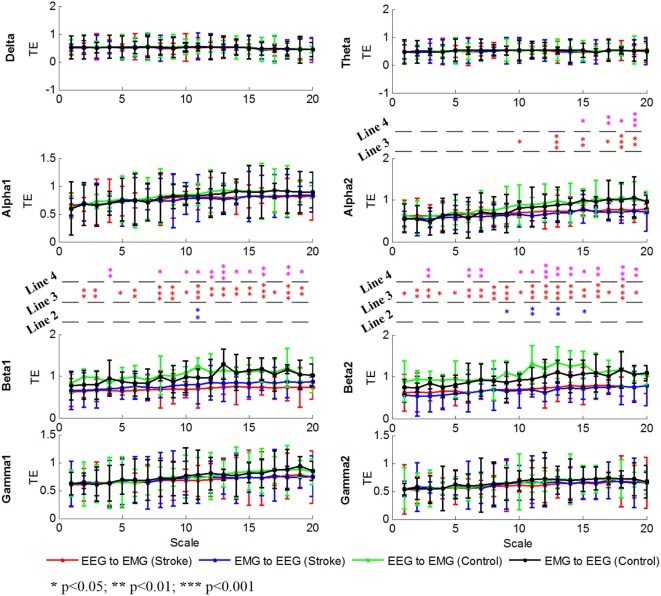
Grand averages of the MSTE values in both descending and ascending directions for all subjects at delta, theta, alpha1, alpha2, beta1, beta2, gamma1, and gamma2 bands, respectively. We denoted the significance with the star mark. *p < 0.05, **p < 0.01, and ***p < 0.001. The word “Line 1” showed the significant level between descending and ascending direction in each time scale for stroke patients, the word “Line 2” was for healthy controls. The Line 3 and Line 4 indicated the significant level between stroke patients and healthy controls at each time scales in descending and ascending directions, respectively.

To further illustrate the differences between stroke patients and healthy controls driving from delta, theta, alpha, beta, or gamma oscillation, we calculated the MSTE values in 8 bands (delta, theta, alpha1, alpha2, beta1, beta2, gamma1, and gamma2) as shown in Figure 5. There were increased trends at the alpha1, alpha2, beta1, and beta2 bands as the scale increased, which was line with the result for the MSTE values across the whole bands in Figure 4. We found that there were no differences at delta, theta, alpha1, gamma1, and gamma2 bands in two directions for stroke patients and healthy controls. There was also no difference between the descending and ascending directions for stroke patients at alpha2, beta1, and beta2 bands, while some differences for healthy controls at beta1 band at scale 11 [*F*(1,14) = 10.90, *p* = 0.008] and beta2 band at scale 15 [*F*(1,14) = 8.46, *p* = 0.019], scale 17 [*F*(1,14) = 9.17, *p* = 0.007], scale 18 [*F*(1,14) = 7.32, *p* = 0.026], and scale 19 [*F*(1,14) = 102.43, *p* = 0.000]. Unlike to the results showed in Figure 5 at scale 1, there were no differences at scale 1 between the descending and ascending directions for healthy controls at all frequency bands. Additionally, significant differences in beta1 and beta2 bands were almost across the entire scales between the stroke patients and healthy controls in both directions. This showed that stroke patients had lower MSTE values at beta1 and beta2 bands in both directions than healthy controls. In this figure, we can find that the differences between the stroke patients and healthy controls which may drive from the beta1 and beta2 band, partly from the alpha2 band. Different from the result in Figure 4, Figure 5 showed us the difference between stroke patients and healthy controls almost across the entire time scales, especially for the descending direction.

## 4 Discussion

As far as we know, the corticomuscular coupling between the brain and the muscles mainly refer to the coupling strength and information flow in single time scale, except for our previous studies (32). Compared to extensive researches on the coupling strength, few studies involve the information flow although it is obvious that the sensorimotor system loop is direction-dependent (21, 22). Witham et al. (24) found that the FCMC in the ascending direction was dominate within the whole beta band compared to that in the descending direction in humans, despite revealed that directed coherence being dominant in the descending direction on monkeys (23). This difference may be due to the differences in sensory feedback between monkeys and humans. Mima et al. (25) reported that FCMC at the 19–30 Hz band from EEG to EMG was significantly larger than that from EMG to EEG. Our research also supports that the EEG beta oscillations propagate bidirectionally between the motor cortex and the corresponding muscle (2, 24, 25, 46). However, in our study, we found that significantly larger single scale-based FCMC in descending direction than in ascending direction, while we found no difference at each frequency band between two directions with time scale 1. Therefore, it is hard to reach a uniform conclusion. Hence, it remains unclear why the FCMC varies between descending and ascending pathways. One possible explanation is that the minor differences in structural anatomy could cause a different projection of activity in the pathways (24). Sensory inputs from muscle spindles ascend through spinal cord to the thalamus and eventually reach the primary somatic sensory cortex (the afferent pathway), and then command outputs from the motor areas in the cerebral cortex to descend through the brain stem to motor neurons of the spinal cord and eventually reach muscle (the efferent pathway). These structural differences may result in the differences between descending and ascending pathways. Another possible influence may come from neurotransmission transition. In contrast to sensory systems which transform physical energy to neural signals, motor systems translate neural signals into contractile forces to produce movements (47).

Compared to the FCMC in healthy controls, a few studies were carried out in stroke patients. In previous studies, only coupling strength was investigated in stroke patients. Though several researches have reported significantly lower corticomuscular coupling for the stroke-affected hand (15–18), there is no any report that points out the difference of the information flow for stroke patients. In our study, we explored the difference between the stroke patients and healthy controls in both directions and found that single scale-based interaction strength in stroke-affected hand was weaker in descending direction than that in healthy controls, and further analysis on the specific bands showed that the decrease mainly drove from the beta2 band. Additionally, we found that stroke patients exhibited no difference between the descending and ascending directions at the whole bands even all specific bands. These differences reveal that interaction connections between the brain and the muscles have been destroyed due to the structural lesion in the cerebral brain. In previous studies, the research has expounded that the reduced neural oscillation after brain injury and the weak cortical-spinal synaptic connection might have influence on the FCMC (17). This is the first study referring to the information flow for stroke patients, and there are still no recorded literatures about the information flow for stroke. We infer the lesions in the brain which also destroy the structure of the pathway providing an approach to transmit information between the brain and the muscles, and decrease the ability to mobilize and activate the related tissues and organs to participate the task, resulting in partly information missing during the transmission processing due to the reduce of the carriers. As a result, the coupling between the brain and the muscle decreased for stroke patients.

Up to date, certain FCMC characteristics over multiple scales are seldom presented in previous literatures which mainly focus on the single-time scale. The sensor-motor system, as a complex and with various structures, involves multi-layer neurotransmission and multi-characteristic interactions. Some researches on EEG or EMG signals have pointed out that complex self-regulating systems operating across multiple spatial and temporal scales can complicate EEG or EMG series at multiple scales (26, 48–50). In our study, multiscale characteristics also exist in sensory-motor system by analyzing the synchronous oscillations between the cerebral cortex and the corresponding muscles. Our previous study have illustrated that the FCMC between the brain cortex and the muscles are multiscale characteristics (32). In this study, we also found healthy controls exhibiting a gradually increasing trend with gradually declining growth rate in both two directions as the scale s increased, and reached a steady state when the scale reached up to 12 even if the scale increased. With respect to why interaction strength will increase as time scale increases, this might be related with the coordination between the brain and the muscles in sensory-motor system, because some studies point out that the coordination functions of the brain can be presented on larger scales on the whole. The sensory input from muscle receptors and the motor command input from the brain can lead to the synchronization oscillations with different strengths and modalities. However, there is no definite explanation for this phenomenon.

The MSTE values in descending direction were higher than that in ascending direction at scale 1, 7, 12, and 14. Further analysis showed that these characteristics in multi-time scale mainly focused on the beta1 band at scale 11 and beta2 band at scale 15, 17, 18, and 19. Our further analysis on specific bands showed that this difference mainly focused on the beta band (beta1 and beta2). Our results reinforce that beta rhythm is primarily rooted in the primary motor cortex (51) and that oscillations in the beta band (15–35 Hz) are associated with controlling and maintaining steady-state force output (6–11). Compared to healthy controls, the FCMC characteristics in multi-time scale for stroke patients were changed. The strengths in both directions were reduced and the gaps between the descending and ascending directions were narrowed over all scales. Further analysis in specific bands showed that the reduced FCMC mainly focused on the alpha2 band at partly high time scales, beta1 band at high time scales and beta2 band at almost all time scales in both directions. This was different from the single scale-based result that there was no difference in each specific band. These differences may drive from complex mix of factors. As our infer in the paragraph above, the lesion in the brain may decrease the ability to mobilize and activate the related tissues and organs to participate the task, resulting in not only losing partly information, but also reducing the dimensionality and complexity of the sensorimotor system. As we see, there were no significant multiscale characteristics for stroke patients. With respect to why significant differences between the stroke patients and healthy controls mainly focused on beta band, it is related to the functions of the beta oscillation which also are illustrated above. For stroke patients, lower stability and poorer performance may result in the weaker coupling at beta band compared to healthy controls. The contents between the motor performance and the FCMC will be studied in the future work.

## 5 Conclusion

In this study, we used the MSTE model to explore the FCMC changes of the inherent directionality and multiscale in sensorimotor systems for stroke patients. Our results showed that the multiscale properties of the FCMC for stroke were changed, the strengths in both directions were reduced and the gaps between the descending and ascending directions were disappeared over all scales. Further analysis in specific bands showed that the reduced FCMC mainly focused on the alpha2 at higher scale, beta1 and beta2, at almost whole scales. This study confirms that the FCMC between the brain and the muscles is capable of multiscale characteristics, and the changes in functional connection for recovered stroke might result from the structural lesion that disrupt coordination, feedback, and information transmission in efferent control and afferent feedback. The study demonstrates for the first time the multiscale characteristics of the FCMC between the brain and the contralateral muscle in both pathways for stroke patients.

## Ethics Statement

All subjects participated according to the declaration of Helsinki and gained consent and approval of the ethical review board of Yanshan University.

## Author Contributions

PX and XC were responsible for the design of the research. XC, YZ, and YC were in-charge of collecting and analyzing the EEG and EMG data. XC, FY, and YZ were responsible for reviewing relevant literatures. LZ and XL provided the physiological mechanism for this discussion. XC, PX, and XL drafted and integrated the manuscript in progress. All authors have read and approved the final manuscript.

## Conflict of Interest Statement

The authors declare that the research was conducted in the absence of any commercial or financial relationships that could be construed as a potential conflict of interest.
